# Endoscopic harvest of sural nerve for grafting in infants with brachial plexus birth injury

**DOI:** 10.1177/17531934221088251

**Published:** 2022-03-28

**Authors:** Willem Pondaag, Justus L. Groen, Martijn J. A. Malessy

**Affiliations:** Department of Neurosurgery/Leiden Nerve Center, Leiden University Medical Center, Leiden, The Netherlands

Dear Editor,

Techniques to harvest the sural nerve (SN) under endoscopic guidance in infants with brachial plexus birth injury (BPBI) have previously been described (Capek et al., 1996). We present two modifications to improve the safety and efficiency of this technique, without the need for any specially designed instruments.

Clarke was the first to use an endoscope for SN harvesting in BPBI with the aid of a specifically designed retractor (Capek et al., 1996). They used prone position to harvest the SNs in both legs, prior to surgical exploration of the brachial plexus, which was done in a supine position. The main downside of their technique is that it does not allow simultaneous exploration of the brachial plexus and SN harvesting due to the need to change patient positioning. In addition, there may not be a need to harvest both SNs in every patient with BPBI. Van Ouwerkerk (1999) used a nerve stripper and a disposable endoscope for this procedure, while others have described a specific endoscopic dissector (Spinks and Adelson, 2009).

We describe a modified technique using a bar mounted on the operating table, a straight-view endoscope, a Kilian nasal speculum (75 mm), a nerve hook (4 mm tip, 90° angle) and a bayonet micro-scissors, all of which are routinely available instruments in an operating theatre.

**Figure 1.**
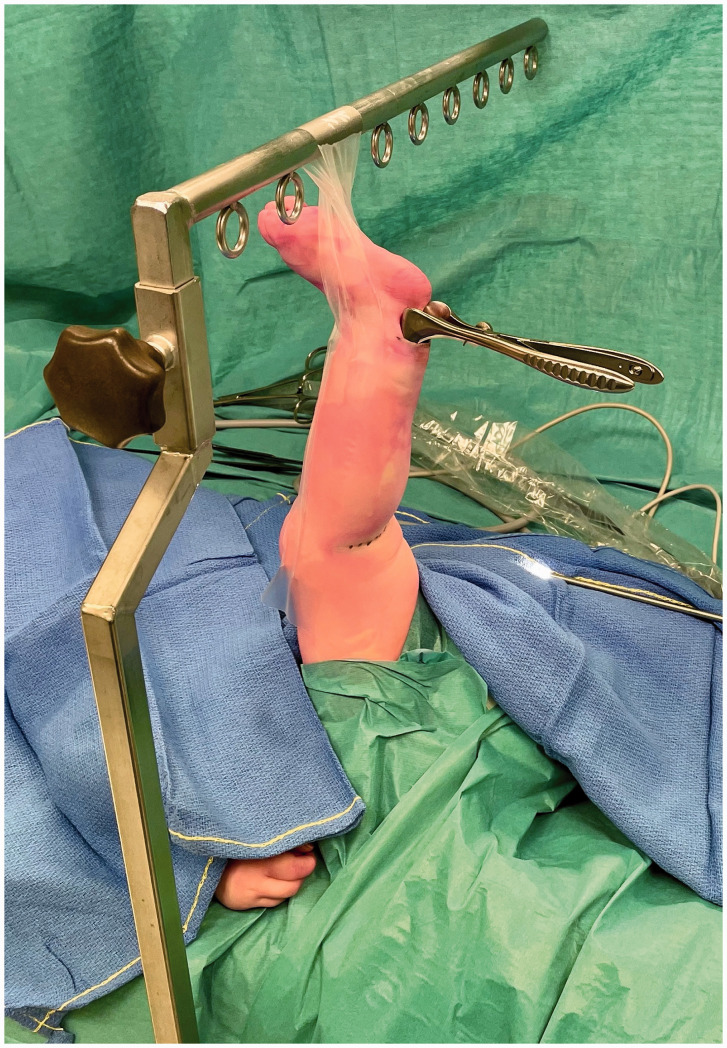
Theatre set-up showing the child in supine position with the left leg suspended from a table-mounted bar (head of the patient is to the left of the picture). The Kilian nasal speculum is seen inserted in the distal incision.

**Figure 2.**
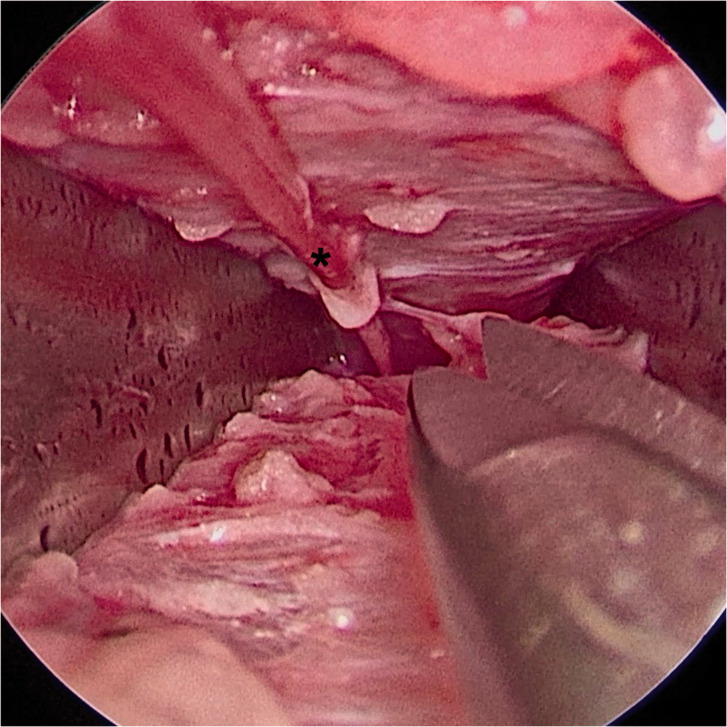
Endoscopic view of the sural nerve (SN) (marked with an asterisk) with its distal portion in the top left of the picture; the blades of the Killian speculum are medial and lateral to the SN. The micro-scissors are shown to illustrate the dimensions.

The child is placed in a supine position and the donor leg for nerve harvesting is hung from a table-mounted bar using adhesive tape with the hip in flexion and knee in full extension (Figure 1). This positioning allows simultaneous access to the brachial plexus and the SN donor site. The bar can also be used at the same time for upward retraction of the clavicle to increase retroclavicular accessibility (Tse et al., 2014). SN is identified through a straight 2 cm skin-crease incision made behind the lateral malleolus and neurolysis is done under loupe magnification using Jameson scissors. After neurolysis of approximately 3 cm of the SN, a Kilian speculum is inserted with the blades medial and lateral to the SN. The speculum can also be used for blunt dissection by opening and closing manoeuvres to create a proper corridor until it can be inserted to its full length. At this point a straight view endoscope is introduced and further neurolysis of the SN is done under endoscopic vision using a combination of blunt dissection with a nerve hook and sharp dissection with bayonet micro-scissors (Figure 2). The SN is freed as far proximal as possible, but at least until it runs under the fascia.

A second 2 cm incision is made in the crease of the popliteal fossa where identification of the proximal SN can be facilitated by gently moving the distal SN. Absence of motor response upon direct electrical stimulation is used for positive SN identification. The tibial and peroneal nerves are identified. The SN is dissected as far proximally as possible and then cut. The SN is then gently lead through the ankle incision and cut as far distally as possible. Both incisions are closed in a subcuticular fashion. A third mid-calf incision may be required for neurolysis at the level where the SN crosses through the fascia if it cannot be reached through the ankle incision.

Since January 2000 we have used this technique to harvest one or two SNs as needed in approximately 400 infants with BPBI. Due to incomplete coding in our database, the exact number of patients or the number of SNs harvested per patient cannot be provided. Our surgical time to harvest one SN graft of 10–13 cm, from incision to wound dressing, has ranged between 35 to 55 minutes. We have found paracetamol to be sufficient in controlling postoperative pain in our series. Surgical scars have been barely visible using skin line incisions and subcuticular closure. Our postoperative complications included one superficial wound infection and one partial palsy of foot extension that resolved spontaneously. None of the patients required conversion to open dissection.

Our modifications of supine positioning of the child allows synchronous brachial plexus exploration and SN nerve harvesting, thus reducing operating time. The decision to harvest one or two SNs can be made after assessment of the severity of the nerve lesion. The use of a Kilian nasal speculum provides an ideal working corridor to neurolyse the SN and helps to minimize the number of skin incisions.
